# Local certification of unitary operations

**DOI:** 10.1038/s41598-024-75148-z

**Published:** 2024-11-04

**Authors:** Ryszard Kukulski, Mateusz Stępniak, Kamil Hendzel, Łukasz Pawela, Bartłomiej Gardas, Zbigniew Puchała

**Affiliations:** 1 Faculty of Physics, Astronomy and Applied Computer Science, Jagiellonian University, ul. Łojasiewicza 11, 30-348 Kraków, Poland; 2 Quantumz.io Sp. z o.o., Puławska 12/3, 02-566 Warsaw, Poland; 3 Institute of Theoretical and Applied Informatics, Polish Academy of Sciences, Bałtycka 5, 44-100 Gliwice, Poland

**Keywords:** Quantum information, Quantum metrology

## Abstract

In this work, we analyze the local certification of unitary quantum channels, which is a natural extension of quantum hypothesis testing. A particular case of a quantum channel operating on two systems corresponding to product states at the input, is considered. The goal is to minimize the probability of the type II error, given a specified maximum probability of the type I error, considering assistance through entanglement with auxiliary systems. Our result indicates connection of the local certification problem with a product numerical range of unitary matrices. We show that the optimal local strategy does not need usage of auxiliary systems and requires only single round of one-way classical communication. Moreover, we compare local and global certification strategies and show that typically local strategies are optimal, yet in some extremal cases, where global strategies make no errors, local ones may fail miserably. Finally, some application for local certification of von Neumann measurements are discussed as well.

## Introduction

In quantum information theory, a well-known problem is the discrimination of states and quantum channels, as solved by Helstrom^1^. This problem involves distinguishing which state or channel from a given pair we are dealing with, based on a prepared measurement. It plays a pivotal role in the comprehension and manipulation of quantum systems. The ensuing step is certification, a process aimed at confirming whether a given hypothesis regarding the state, channel, or measurement holds true; this is achieved by contrasting it with an alternative hypothesis. Certification safeguards the integrity and reliability of quantum operations, rendering it indispensable for quantum computing and communications. The mathematical explanation of the *modus operandi* of quantum computers involves the use of quantum operations and channels. This paper focuses on the local certification of unitary operations, a technique which is crucial for enhancing quantum computing applications, and developing quantum algorithms and error correction strategies. This certification is useful for benchmarking quantum devices, thus steering the progression of quantum algorithm design.

Great deal of work has been done in the domain of local certification and distinguishability of quantum states. The research were focused on presenting conditions for a finite set of orthogonal quantum states to be distinguishable by local operations^2–6^. It has been noticed, that local certification strategies not always are as powerful as global strategies involving usage of a quantum entanglement. Many examples of sets of orthogonal quantum states that cannot be perfectly certified were discovered in the literature^7,8^; also in the domain of mixed quantum states^9^.

The following up research about local discrimination of unitary channels has been built upon the results concerning quantum states. It was observed that there exist discrimination problems for which local procedures may be optimal as well as there exist problems for which they perform poorly^10^. Some conditions concerning optimal local discrimination strategies were already proposed too^11^. Eventually, couple of works focused on many copies scenario, were it was shown that any two different unitary operations acting on an arbitrary multipartite quantum system can be perfectly distinguishable by local operations and classical communication when a finite number of runs^12–14^. However, in the literature, the problem of certification of unitary channels has not been yet considered. Taking up this challenge, in this work we explore a scenario where two parties, having access to a shared quantum unitary channel, engage in its certification. We compare local certification strategies with global ones and find the optimal and resource efficient local certification strategies.

Certification is closely related to statistical hypothesis testing, which is a fundamental concept in statistical decision theory^15^. We consider a system with two hypotheses: the null hypothesis ($H_0$) and the alternative hypothesis ($H_1$). The null hypothesis intuitively corresponds to a promise about the system given by its creator. By performing a test, we decide which hypothesis to accept as true. A type I error occurs if we reject the null hypothesis when it is true. The probability of this error occurring is called the level of significance. On the other hand, a type II error occurs when we accept the null hypothesis, even though it is false. We want to minimize the type II error given an assumed level of significance. This approach is commonly referred to as certification. It turns out that the concept of the numerical range of a matrix is an useful tool in such issues. The numerical range provides insights into the spectral and structural properties of matrices, making it important in quantum mechanics^16^.

## Preliminaries

In this section, we elucidate the foundational principles underlying the certification processes under consideration.

### Notation

In this work, we will encounter the notation of quantum states, quantum measurements and quantum channels. In order to set notation, we mention them here briefly (see^17^ for more detailed explanation).

We say that operator $\rho$ represents a quantum state defined on a system of dimension *d* if this operator is positive semidefinite ($\rho \ge 0$) with unit trace ($\mathrm{{tr}}\rho = 1$). An linear map $\Psi$ will be called quantum channel if it is completely positive and trace preserving map (CPTP). We will consider a special family of quantum channels known as unitary channels. For an unitary matrix *U* we define unitary quantum channel $\Psi _U$ by

1 $$\begin{aligned} \Psi _U(\rho )= U \rho U^\dagger , \end{aligned}$$

where $\rho$ is an input quantum state $\rho$.

In a finite-dimensional case, quantum measurement, also called as positive operator-valued measure (POVM), is be represented by a set of positive semidefinite operators $\Omega = \{\Omega _i\}_i$ (also known as effects), such that $\sum _i \Omega _i = \mathrm{1\hspace{-0.9mm}l}$. According to the Born rule, for a given state $\rho$ the probability of obtaining the measurement outcome *i* is given by $p_i = \mathrm{{Tr}}\rho \Omega _i$. The special subclass of quantum measurements consists of Von Neumann measurements. They fulfill the additional requirement that all effects $\Omega _i$ are rank-one projectors. Hence, for a von Neumann measurement acting on a state of dimension *d* there are exactly *d* effects $\Omega _i$ which are pairwise orthogonal. This simple observation allows us to parameterize a *d*-dimensional von Neumann measurement using a unitary matrix *U*, $P_U = \{U|i\rangle \!\langle i|U^\dagger \}_{i=1}^d$. As a shorthand notation, we will write ${|{u_i}\rangle } {:}{=}U{|{i}\rangle }$. We can associate a measure-and-prepare channel with a von Neumann measurement

2 $$\begin{aligned} P_U(\rho ) = \sum _{i=1}^{d} {\langle {u_i}|} \rho {|{u_i}\rangle } |i\rangle \!\langle i|. \end{aligned}$$

Finally, in this work we will consider the family of bipartite quantum channels and measurements that can be realized by using only local operations and classical communication (LOCC) between two involved parties, say Alice and Bob. Let us assume that Alice has access to a system $\mathcal {A}$ and Bob to a system $\mathcal {B}$. Then, we say that bipartite quantum channel $\Psi$ (or measurement $\Omega$) is LOCC with respect to the partition $\mathcal {A}:\mathcal {B}$, if it can be realized by using local quantum operations on $\mathcal {A}$ and $\mathcal {B}$ separately, and by sharing classical information between $\mathcal {A}$ and $\mathcal {B}$. Such operations will be used notoriously in this work to prepare input quantum states and POVMs without creating quantum entanglement between involved parties.

### Numerical ranges of a matrix

In the context of certifying unitary channels, the numerical range and the product numerical range play a pivotal role. The *numerical range* of a square matrix *X* of size *d*, is defined as a subset of the complex plane:

3 $$\begin{aligned} W(X)= \{ {\langle {\psi }|} X {|{\psi }\rangle } : \langle \psi | \psi \rangle = 1, {|{\psi }\rangle } \in \mathbb {C}^d \}. \end{aligned}$$

The set *W*(*X*) is compact and convex; an in depth discussion of its properties and application can be found in^18,19^.

The *product numerical range* of a square matrix *X* of size $d_1 \cdot d_2$ with the partition $d_1: d_2$ is defined as:

4 $$\begin{aligned} \begin{aligned} W^{\otimes }_{d_1:d_2}(X)&= \{ ({\langle {\psi _1}|} \otimes {\langle {\psi _2}|}) X ({|{\psi _1}\rangle } \otimes {|{\psi _2}\rangle }) : \\&\phantom { = } \langle \psi _1| \psi _1\rangle = 1, \langle \psi _2| \psi _2\rangle = 1, \\&\phantom { = } {|{\psi _1}\rangle } \in \mathbb {C}^{d_1} , {|{\psi _2}\rangle } \in \mathbb {C}^{d_2} \}. \end{aligned} \end{aligned}$$

The core properties of this object are described in^20^.

### Operational scenario

**Fig. 1:**
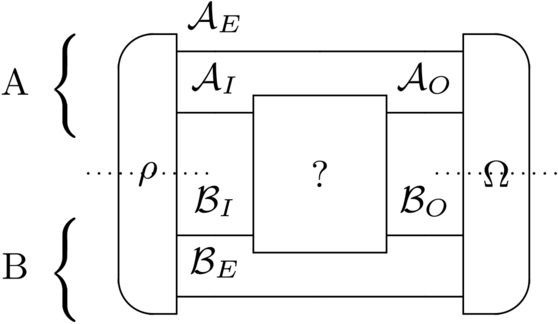
Schematic representation of the operational scenario for unitary channel certification. Alice (**A**) and Bob (**B**) can prepare the initial state $\rho$ and the final measurement $\Omega$ by using LOCC operations to certificate if $? = \Psi _U$ or $? = \Psi _V$.

In the certification of a unitary channel, two parties, Alice and Bob, have an access to a quantum unitary channel. They have knowledge that the given channel is one of two possible unitary channels: $\Psi _U$, which will be identified with $H_0$ hypothesis or $\Psi _V$, which will be identified with $H_1$ hypothesis. Alice and Bob do not know which one is provided to them. Their goal is to find the best strategy to certificate that the given unknown channel is $\Psi _U$ and detect whenever the unknown operation is $\Psi _V$. More precisely, given the level of significance $\delta \ge 0$ as a parameter, they want to minimize the probability of making type II error provided the probability of making type I error is not greater than $\delta$.

The unknown operation has two inputs on the spaces $\mathcal {A}_I$ and $\mathcal {B}_I$, and two outputs on the spaces $\mathcal {A}_O$ and $\mathcal {B}_O$ (see Fig. 1). Alice has an access to spaces $\mathcal {A}_I$ and $\mathcal {A}_O$ and auxiliary space $\mathcal {A}_E$, while Bob to $\mathcal {B}_I$ and $\mathcal {B}_O$ and auxiliary system $\mathcal {B}_E$, respectively. To produce an input state $\rho$ Alice and Bob are limited to use LOCC operations with respect to the partition $\mathcal {A}_I \otimes \mathcal {A}_E: \mathcal {B}_I \otimes \mathcal {B}_E$. We will write in short that $\rho \in \texttt{LOCC}$. Similarly, to produce a POVM $\Omega = \{\Omega _0, \Omega _1\}$ Alice and Bob are limited to use LOCC operations with respect to the partition $\mathcal {A}_O \otimes \mathcal {A}_E: \mathcal {B}_O \otimes \mathcal {B}_E$. We will use the notation $\Omega \in \texttt{LOCC}$. Based on the results (classical labels) of their measurements, they decide, which unitary channel they are dealing with: label 0 associated with the effect $\Omega _0$ indicates $H_0$, while label 1 associated with the effect $\Omega _1$ indicates $H_1$.

## Main results

### Unitary channel certification

In this section we will state the solution to the problem introduced in the Section 2.3. Let $\mathcal {A}_I = \mathcal {A}_O = \mathbb {C}^{d_1}$, $\mathcal {B}_I = \mathcal {B}_O = \mathbb {C}^{d_2}$. We are given two unitary matrices *U* and *V* of size $d_1 \cdot d_2$ and consider two hypotheses:

- $H_0$: The operation is $\Psi _U$.
- $H_1$: The operation is $\Psi _V$.

As mentioned previously (see also Fig. 1), Alice and Bob can prepare the input state $\rho$ and the measurement $\Omega = \{\Omega _0, \Omega _1\}$ by using LOCC operations with respect to the partitions $\mathcal {A}_I \otimes \mathcal {A}_E: \mathcal {B}_I \otimes \mathcal {B}_E$ and $\mathcal {A}_O \otimes \mathcal {A}_E : \mathcal {B}_O \otimes \mathcal {B}_E$, respectively. The dimension of spaces $\mathcal {A}_E$ and $\mathcal {B}_E$ are arbitrary. Alice and Bob accept accept the null hypothesis if the measurement result is $\Omega _0$, otherwise, they reject it. Thus, we arrive at the following formulas for the probabilities of type I and type II errors:

5 $$\begin{aligned} \begin{aligned} p_{\textrm{I}}(\Omega , \rho )&= \mathrm{{tr}}\left( \Omega _1 (\Psi _U \otimes \mathrm{1\hspace{-0.9mm}l}_{\mathcal {A}_E \otimes \mathcal {B}_E})(\rho )\right) , \\ p_{\textrm{II}}(\Omega , \rho )&= \mathrm{{tr}}\left( \Omega _0 (\Psi _V \otimes \mathrm{1\hspace{-0.9mm}l}_{\mathcal {A}_E \otimes \mathcal {B}_E})(\rho )\right) . \end{aligned} \end{aligned}$$

Certification requires minimizing $p_{\textrm{II}}$ under the condition $p_{\textrm{I}}\le \delta$, where $\delta$ is the desired significance level. It leads to the following optimization problem:

6 $$\begin{aligned} p_{\textrm{II}}(U, V) {:}{=}\min \{ p_{\textrm{II}}(\Omega , \rho ): p_{\textrm{I}}(\Omega , \rho ) \le \delta , \rho , \Omega \in \texttt{LOCC} \}. \end{aligned}$$

The remainder of this section is devoted to proving the following theorem. This result shows the relation between two-party certification and the product numerical range.

#### Theorem 1

*Consider the problem of two-point certification of unitary channels with hypotheses*

- $H_0$*: The operation is* $\Psi _U$.
- $H_1$*: The operation is* $\Psi _V$.

*defined as in Section* 3.1*for unitary matrices* *U* *and* *V* *of size* $d_1 \cdot d_2$*, and statistical significance* $\delta \in [0,1]$*. Let z be the euclidean distance between* 0 *and* $W^{\otimes }_{d_1:d_2}(V^\dagger U)$*, that is*

7 $$\begin{aligned} z {:}{=}\min \{ |x|: x \in W^{\otimes }_{d_1:d_2}(V^\dagger U) \}. \end{aligned}$$

*Then, for the most powerful test utilizing LOCC operations, the probability of the type II error yields*

8 $$\begin{aligned} \begin{aligned} p_{\textrm{II}}(U, V)= {\left\{ \begin{array}{ll} 0, & z \le \sqrt{\delta }, \\ \left( z\sqrt{1-\delta } - \sqrt{1 - z^2}\sqrt{\delta }\right) ^2, & z > \sqrt{\delta }. \end{array}\right. } \end{aligned} \end{aligned}$$

#### Proof

By utilizing local quantum operations and classical communication, Alice and Bob can prepare any separable quantum state $\rho = \sum _j p_j |a_j\rangle \!\langle a_j| \otimes |b_j\rangle \!\langle b_j|,$ where $|a_j\rangle \!\langle a_j|$ are defined on $\mathcal {A}_I \otimes \mathcal {A}_E$ and $|b_j\rangle \!\langle b_j|$ on $\mathcal {B}_I \otimes \mathcal {B}_E$.

Let us assume that Alice and Bob take a product state, that is $\rho = |a,b\rangle \!\langle a,b| = |a\rangle \!\langle a| \otimes |b\rangle \!\langle b|$. Then, depending which hypothesis is true, we obtain the one of the following pure states

9 $$\begin{aligned} \begin{aligned} H_0:&\quad |h_0\rangle \!\langle h_0| {:}{=}(\Psi _U \otimes \mathrm{1\hspace{-0.9mm}l}_{\mathcal {A}_E \otimes \mathcal {B}_E})(\rho ),\\ H_1:&\quad |h_1\rangle \!\langle h_1| {:}{=}(\Psi _V \otimes \mathrm{1\hspace{-0.9mm}l}_{\mathcal {A}_E \otimes \mathcal {B}_E})(\rho ). \end{aligned} \end{aligned}$$

We consider two cases. If $|\langle h_0| h_1\rangle | \le \sqrt{\delta }$, then according to^21^, Theorem 1 & Corollary 1 the best pure states certification strategy $\widetilde{\Omega }$ with significance level $\delta$ is of the form: $\widetilde{\Omega } = \{\widetilde{\Omega _0}, \widetilde{\Omega _1}\}$, where $\widetilde{\Omega _0} = |\omega \rangle \!\langle \omega |$ for ${|{\omega }\rangle } = \frac{{|{\widetilde{\omega }}\rangle }}{\Vert \widetilde{\omega } \Vert }$ and ${|{\widetilde{\omega }}\rangle } = {|{h_0}\rangle } - \langle h_1| h_0\rangle {|{h_1}\rangle }$. The operator $|\omega \rangle \!\langle \omega |$ satisfies $|\langle h_1| \omega \rangle |^2 = 0$ and $|\langle h_0| \omega \rangle |^2 \ge 1 - \delta$. Therefore, the states $|h_1\rangle \!\langle h_1|$ and $|\omega \rangle \!\langle \omega |$ are orthogonal and we can find a LOCC measurement $\Omega = \{\Omega _0, \Omega _1\}$^22^, such that $\mathrm{{tr}}(\Omega _0 |\omega \rangle \!\langle \omega |) = 1$ and $\mathrm{{tr}}(\Omega _1 |h_1\rangle \!\langle h_1|) = 1$. Alice and Bob choose $\Omega$ as their measurement and achieve $p_{\textrm{I}}(\Omega , \rho ) = \mathrm{{tr}}(\Omega _1 |h_0\rangle \!\langle h_0|) \le 1 - \mathrm{{tr}}(|\omega \rangle \!\langle \omega ||h_0\rangle \!\langle h_0|) \le \delta$ and $p_{\textrm{II}}(\Omega , \rho ) = 1 - \mathrm{{tr}}(\Omega _1 |h_1\rangle \!\langle h_1|) = 0$, which is the optimal solution in that case.

If $|\langle h_0| h_1\rangle | > \sqrt{\delta }$, then according to^21^, Theorem 1 & Corollary 1 the best pure states certification strategy $\widetilde{\Omega }$ with significance level $\delta$ is of the form: $\widetilde{\Omega } = \{\widetilde{\Omega _0}, \widetilde{\Omega _1}\}$, where $\widetilde{\Omega _0} = |\omega \rangle \!\langle \omega |$ for ${|{\omega }\rangle } = \sqrt{1-\delta }\frac{\langle h_0| h_1\rangle }{|\langle h_0| h_1\rangle |} {|{h_0}\rangle } - \sqrt{\delta } {|{h_0^\perp }\rangle }$ and ${|{h_0^\perp }\rangle } = {|{\widetilde{h_0^\perp }}\rangle } / \Vert \widetilde{h_0^\perp }\Vert$, where ${|{\widetilde{h_0^\perp }}\rangle } = {|{h_1}\rangle } - \langle h_0| h_1\rangle {|{h_0}\rangle }$. The operator $|\omega \rangle \!\langle \omega |$ satisfies $|\langle h_1| \omega \rangle |^2 = (\sqrt{1-\delta } |\langle h_0| h_1\rangle | - \sqrt{\delta } \sqrt{1 - |\langle h_0| h_1\rangle |^2})^2$ and $|\langle h_0| \omega \rangle |^2 = 1 - \delta$. Here, for the orthogonal states $|\omega \rangle \!\langle \omega |$ and $|\omega ^\perp \rangle \!\langle \omega ^\perp |$, where ${|{\omega ^\perp }\rangle } = \sqrt{\delta } {|{h_0}\rangle } + \sqrt{1-\delta } \frac{\langle h_1| h_0\rangle }{|\langle h_0| h_1\rangle |} {|{h_0^\perp }\rangle }$ we can find a LOCC measurement $\Omega = \{\Omega _0, \Omega _1\}$^22^, such that $\mathrm{{tr}}(\Omega _0 |\omega \rangle \!\langle \omega |) = 1$ and $\mathrm{{tr}}(\Omega _1 |\omega ^\perp \rangle \!\langle \omega ^\perp |) = 1$. Alice and Bob choose $\Omega$ as their measurement and achieve $p_{\textrm{I}}(\Omega , \rho ) = \mathrm{{tr}}(\Omega _1 |h_0\rangle \!\langle h_0|) \le 1 - \mathrm{{tr}}(|\omega \rangle \!\langle \omega ||h_0\rangle \!\langle h_0|) = \delta$. To calculate $p_{\textrm{II}}(\Omega , \rho )$ notice that ${|{h_1}\rangle }$ is spanned in the basis ${|{\omega }\rangle }, {|{\omega ^\perp }\rangle }$, that is ${|{h_1}\rangle } = c_1 {|{\omega }\rangle } + c_2 {|{\omega ^\perp }\rangle }$. Therefore, we get $p_{\textrm{II}}(\Omega , \rho ) = \mathrm{{tr}}(\Omega _0 |h_1\rangle \!\langle h_1|) = |c_1|^2\mathrm{{tr}}(\Omega _0 |\omega \rangle \!\langle \omega |) = |c_1|^2$, where we used $\Omega _0{|{\omega ^\perp }\rangle } = {|{\omega ^\perp }\rangle } - \Omega _1{|{\omega ^\perp }\rangle } = {|{\omega ^\perp }\rangle } - {|{\omega ^\perp }\rangle } = 0$. Eventually, $p_{\textrm{II}}(\Omega , \rho ) =|c_1|^2 = |\langle h_1| \omega \rangle |^2$, which is the optimal solution in that case.

We have showed that the optimal measurement $\Omega$ for product state $\rho$ gives

10 $$\begin{aligned} p_{\textrm{II}}(\Omega , \rho ) = {\left\{ \begin{array}{ll} 0, & |x| \le \sqrt{\delta }, \\ (\sqrt{1-\delta } |x| - \sqrt{\delta } \sqrt{1 - |x|^2})^2, & |x| > \sqrt{\delta }, \end{array}\right. } \end{aligned}$$

where $x {:}{=}\langle h_1| h_0\rangle = {\langle {a, b}|} \left( V^\dagger U \otimes \mathrm{1\hspace{-0.9mm}l}_{\mathcal {A}_E \otimes \mathcal {B}_E}\right) {|{a,b}\rangle }.$ As the function $|x| \mapsto p_{\textrm{II}}(\Omega , \rho )$ is non-decreasing, Alice and Bob choose ${|{a,b}\rangle }$ that minimizes |*x*|.

Observe, the choice of the optimal product input state $\rho = |a,b\rangle \!\langle a,b|$ is independent of the significance level $\delta$. Therefore, no separable state $\rho$ will provide better result than product state.

Finally, let us assume that $\rho = |a_0, b_0\rangle \!\langle a_0, b_0|$ is the optimal product input state - it minimizes $|a,b\rangle \!\langle a,b| \mapsto |{\langle {a, b}|} \left( V^\dagger U \otimes \mathrm{1\hspace{-0.9mm}l}_{\mathcal {A}_E \otimes \mathcal {B}_E}\right) {|{a,b}\rangle }|.$ Let $\mathcal {A}_E = \mathbb {C}^{e_1}$ and $\mathcal {B}_E = \mathbb {C}^{e_2}$. Using the result from^13^, Lemma 2 we get

11 $$\begin{aligned} \begin{aligned}&{\langle {a_0, b_0}|} \left( V^\dagger U \otimes \mathrm{1\hspace{-0.9mm}l}_{\mathcal {A}_E \otimes \mathcal {B}_E}\right) {|{a_0,b_0}\rangle } \\ \in&W^{\otimes }_{d_1e_1:d_2e_2}(V^\dagger U \otimes \mathrm{1\hspace{-0.9mm}l}_{\mathcal {A}_E \otimes \mathcal {B}_E})\\ =&W^{\otimes }_{d_1:d_2}(V^\dagger U). \end{aligned} \end{aligned}$$

Hence, there is a quantum state $|A_0\rangle \!\langle A_0|$ defined on the space $\mathcal {A}_I$ and a quantum state $|B_0\rangle \!\langle B_0|$ on the space $\mathcal {B}_I$, such that ${\langle {a_0, b_0}|} \left( V^\dagger U \otimes \mathrm{1\hspace{-0.9mm}l}_{\mathcal {A}_E \otimes \mathcal {B}_E}\right) {|{a_0,b_0}\rangle } = {\langle {A_0, B_0}|} V^\dagger U {|{A_0,B_0}\rangle }.$ Eventually, we observe that to minimize |*x*| Alice and Bob do not need to use auxiliary systems $\mathcal {A}_E$ and $\mathcal {B}_E$ and the optimal state can be chosen as $\rho = |A_0,B_0\rangle \!\langle A_0,B_0|$, which is defined on $\mathcal {A}_I \otimes \mathcal {B}_I$. Hence, for $z = \min \{ \left| {\langle {a, b}|} V^\dagger U {|{a,b}\rangle } \right| : \langle a| a\rangle = \langle b| b\rangle = 1,{|{a}\rangle } \in \mathbb {C}^{d_1}, {|{b}\rangle } \in \mathbb {C}^{d_2} \} = \min \{|x|: x \in W^{\otimes }_{d_1:d_2}(V^\dagger U) \}$ we achieve the desired result

12 $$\begin{aligned} \begin{aligned} p_{\textrm{II}}(U, V)= {\left\{ \begin{array}{ll} 0, & z \le \sqrt{\delta }, \\ (\sqrt{1-\delta } z - \sqrt{\delta } \sqrt{1 - z^2})^2, & z > \sqrt{\delta }. \end{array}\right. } \end{aligned} \end{aligned}$$

$$\square$$

### Optimal certification strategy

The proof of Theorem 1 provides insight of the best certification strategy that Alice and Bob can utilize. Starting from the input state $\rho \in \texttt{LOCC}$, we see that Alice does not have to create any entanglement between $\mathcal {A}_I$ and $\mathcal {A}_E$, what is more - she does not need to use auxiliary system $\mathcal {A}_E$ at all. The same holds for Bob’s systems. That optimal input state $\rho = |a,b\rangle \!\langle a,b|$ defined on $\mathcal {A}_I \otimes \mathcal {B}_I$ is pure and product as well it minimizes $|{\langle {a,b}|} V^\dagger U {|{a,b}\rangle }|$. It is independent from the significance level $\delta$.

**Fig. 2:**
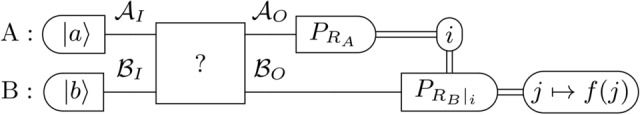
Schematic representation of the optimal operational scenario for unitary channel certification. Alice (**A**) prepares the initial pure state $|a\rangle \!\langle a|$ and Bob (**B**) prepares $|b\rangle \!\langle b|$. The final measurement consists of Alice preparing $P_{R_A}$ and sending the result label *i* to Bob, who prepares the measurement $P_{R_B|_i}$. The post-processed label $j \mapsto f(j) \in \{0,1\}$ of Bob’s measurement certificate if $? = \Psi _U$ or $? = \Psi _V$.

The explicit form of the optimal measurement $\Omega \in \texttt{LOCC}$ is more complicated and relies heavily on the construction provided in^22^ and the proof of Theorem 1. Also, its description changes with $\delta$. Nevertheless, from the operational point of view, $\Omega$ can be simply realized by Alice and Bob. One party, let’s say Alice, prepares an appropriate Von Neumann measurement $P_{R_A}$ on system $\mathcal {A}_O$, where $R_A$ is an unitary rotation and sends the measurement result *i* as a classical information to Bob. Then, he prepares a Von Neumann measurement $P_{R_B |_i}$ on $\mathcal {B}_O$, conditioned on the information gained from Alice, where $R_B |_i$ is an unitary rotation. Bob’s measurement result *j* after classical post-processing $j \mapsto f(j) \in \{0,1\}$ indicates if the hypothesis $H_0$ should be accepted or rejected. We provide a schematic representation of the optimal strategy in Fig. 2.

### Local vs global certification of unitary channels

In the case where a single party controls both inputs and outputs, the party can create entanglement between compound systems $\mathcal {A}_I \otimes \mathcal {A}_E$ and $\mathcal {B}_I \otimes \mathcal {B}_E$ (similarly between $\mathcal {A}_O \otimes \mathcal {A}_E$ and $\mathcal {B}_O \otimes \mathcal {B}_E$). In other words, the input state $\rho$ can be chosen arbitrarily, also the measurement $\Omega = \{\Omega _0, \Omega _1\}$. The certification result $p_{\textrm{II}}^*(U, V)$ is expressed as^21^:

13 $$\begin{aligned} \begin{aligned} p_{\textrm{II}}^*(U, V)= {\left\{ \begin{array}{ll} 0, & v \le \sqrt{\delta }, \\ \left( v\sqrt{1-\delta } - \sqrt{1 - v^2}\sqrt{\delta }\right) ^2, & v > \sqrt{\delta }, \end{array}\right. } \end{aligned} \end{aligned}$$

where

14 $$\begin{aligned} v {:}{=}\min \{ |x|: x \in W(V^\dagger U) \}. \end{aligned}$$

As we can see, the certification results differ in local and global scenarios. Observe, that both results depend on the product $V^\dagger U$, hence, the results are unitarily invariant and we may assume that $V = \mathrm{1\hspace{-0.9mm}l}$ for the remainder of this section. In the global case we are interested in computing the distance $v( U) {:}{=}\min \{ |x|: x \in W(U) \}$, while for the local case we compute the distance $z_{d_1:d_2}(U) {:}{=}\min \{ |x|: x \in W^{\otimes }_{d_1:d_2}(U) \}$. The forthcoming comparison of $z_{d_1:d_2}(U)$ and *v*(*U*) will enable a comparative analysis between single-party and two-party certification scenarios.

From the definition, for all unitary matrices *U* of size $d_1 \cdot d_2$ it holds $v(U) \le z_{d_1:d_2}(U)$. The questions arise, can *v*(*U*) be strictly lower than $z_{d_1:d_2}(U)$ and how much lower it can be?

Let $$d = d_1 = d_2$$. Define

15 $$\begin{aligned} U = \mathrm{1\hspace{-0.9mm}l}_{d^2} - \frac{2}{d} |\mathrm{1\hspace{-0.9mm}l}_d\rangle \!\langle \mathrm{1\hspace{-0.9mm}l}_d|, \end{aligned}$$

where ${|{\mathrm{1\hspace{-0.9mm}l}_d}\rangle } = \sum _{i=1}^d {|{i,i}\rangle }$. Observe that the eigenvalues of *U* are $\pm 1$, so $v(U) = 0$ and therefore, $p_{\textrm{II}}^*(U, \mathrm{1\hspace{-0.9mm}l}_{d^2}) = 0$. On the other hand, for any normed vectors ${|{\psi _1}\rangle }, {|{\psi _2}\rangle } \in \mathbb {C}^d$ we have

16 $$\begin{aligned} \left| {\langle {\psi _1, \psi _2}|} U {|{\psi _1, \psi _2}\rangle } \right| = \left| 1 - \frac{2}{d} |\langle \psi _1| \overline{\psi _2}\rangle |^2 \right| \ge \frac{d-2}{d}, \end{aligned}$$

where to saturate the last inequality we take ${|{\psi _1}\rangle } = {|{\psi _2}\rangle } = {|{1}\rangle }$. That means, $z_{d_1:d_2}(U) = (d-2)/d$ and when the local dimension goes to infinity, $d \rightarrow \infty$, then we have $p_{\textrm{II}}(U, \mathrm{1\hspace{-0.9mm}l}_{d^2}) \rightarrow 1 - \delta$.

We showed that in the extremal case, local and global strategies differ significantly. But what about a typical case? For the incoming analysis we assume that *U* is Haar-random unitary matrix^23^. We have the following theorem.

#### Theorem 2

*Let* *U* *be a Haar-random unitary matrix of size* $d_1 \cdot d_2$*. For large enough product* $d_1d_2$ *we have*

17 $$\begin{aligned} \mathbb {P}\left( z_{d_1:d_2}(U) = 0 \right) \ge 1 - \exp \left( -\frac{\log 2}{2} \max (d_1^2, d_2^2)\right) . \end{aligned}$$

#### Proof

Without loss of the generality let us assume that $d_2 \ge d_1$. For a fixed state ${|{1}\rangle } \in \mathbb {C}^{d_1}$ denote $M = ({\langle {1}|} \otimes \mathrm{1\hspace{-0.9mm}l}) U ({|{1}\rangle } \otimes \mathrm{1\hspace{-0.9mm}l})$. We have then $W\left( M\right) \subset W^{\otimes }_{d_1:d_2}(U)$. As *U* is Haar distributed the matrix *M* has the same distribution as *VM*, where *V* is a Haar-random unitary matrix of size $d_2$ independent of *U*. The matrix *M* is a truncation of *U*, hence, almost surely it has full rank^24^. Continuing the reasoning, if $M = U_M Q_M$ is the polar decomposition of *M*, then *VM* has the same distribution as $VQ_M$. Let $\lambda _i$ be eigenvalues of *V* with corresponding eigenvectors ${|{x_i}\rangle }$. Then ${\langle {x_i}|}VQ_M{|{x_i}\rangle } = \lambda _i {\langle {x_i}|}Q_M{|{x_i}\rangle } \in W(VQ_M)$ for each *i*, where almost surely ${\langle {x_i}|}Q_M{|{x_i}\rangle } > 0$. Therefore, if $0 \in W(V)$, then there is a probability vector $(p_i)_i$ such that $\sum _i \lambda _i p_i = 0$. Let us define a probability vector $(q_i)_i$ given by $q_i = \frac{p_i}{{\langle {x_i}|}Q_M{|{x_i}\rangle }} \left( \sum _j \frac{p_j}{{\langle {x_j}|}Q_M{|{x_j}\rangle }} \right) ^{-1}$. We obtain $\sum _i q_i \lambda _i {\langle {x_i}|}Q_M{|{x_i}\rangle } = 0$ which implies $0 \in W(VQ_M)$. Combining all together we get

18 $$\begin{aligned} \begin{aligned} \mathbb {P}\left( z_{d_1:d_2}(U) = 0 \right)&\ge \mathbb {P}\left( 0 \in W(M) \right) = \mathbb {P}\left( 0 \in W(VQ_M) \right) \\ &\ge \mathbb {P}\left( 0 \in W(V)\right) \ge 1 - \exp \left( -\frac{\log 2}{2} d_2^2\right) , \end{aligned} \end{aligned}$$

where the last inequality was proven in^25^, Proposition 19 for large enough $d_2$. $\square$

According to Theorem 2, when we are dealing with high-dimensional unitary channels $\Psi _U$ and $\Psi _V$, most of them can be perfectly certified. What is more, the optimal strategy is local, uses only once one-way classical communication channel and does not need auxiliary systems (see Fig. 2). Such strategies are the most desirable in terms of used resources such as quantum entanglement^26^.

In this section, we learned that the gap between *v*(*U*) and $z_{d_1:d_2}(U)$ may be huge in extremal cases. Also, we observed that typically in high dimensions both quantities are equal to zero. We provide more examples comparing local and global strategies in the Supplementary Material.

## Application to Von Neumann measurement certification

In the domain of von Neumann measurements certification, we are given two unitary matrices *U* and *V* of size $d_1 \cdot d_2$ and consider two hypotheses:

- $H_0$: The operation is $P_U$.
- $H_1$: The operation is $P_V$.

The operational paradigm is similar, yet it harbors a critical distinction: the output transitions to a classical domain. Upon executing a joint measurement on their respective quantum states, a classical label *i* is generated. This label, mutually acknowledged by Alice and Bob, serves as the foundational element for subsequent certification processes. Thereafter, both parties then measure their auxiliary systems, guided by the known label *i*, to ascertain whether the joint measurement was null of the alternative hypothesis. As in the unitary channel certification Alice and Bob strategy is similar (see Fig. 1). They can prepare the input state $\rho \in \texttt{LOCC}$ and the measurement $\Omega = \{\Omega _0, \Omega _1\} \in \texttt{LOCC}$ while having access to auxiliary systems $\mathcal {A}_E$ and $\mathcal {B}_E$ of arbitrary dimension. Let $\widetilde{p_{\textrm{II}}}(U, V)$ indicates minimized probability of type II error under the condition that $\delta$ is given significance level for the introduced certification scheme of Von Neumann measurements. Then, we summarize our findings with the following proposition:

### Proposition 3

*Consider the problem of two-point certification of Von Neumann measurements defined for unitary matrices* *U* *and* *V* *of size* $d_1 \cdot d_2$*, and statistical significance* $\delta \in [0,1]$ *with hypotheses*

- $H_0$*: The operation is* $P_U$.
- $H_1$*: The operation is* $P_V$.

*The most powerful test utilizing LOCC operations provides*

19 $$\begin{aligned} \begin{aligned} \widetilde{p_{\textrm{II}}}(U, V) \ge \max \{&p_{\textrm{II}}(UE, VF): E, F \text{ is } \text{ unitary } \\&\text{ and } \text{ diagonal }\}. \end{aligned} \end{aligned}$$

### Proof

Observe that action of each von Neumann measurement $P_U$, can be expressed as $P_U = \Delta \Psi _{{(UE)}^{\dag }}$. Here, $\Delta$ is the completely dephasing channel $\Delta (X) = \sum _i {\langle {i}|} X {|{i}\rangle } |i\rangle \!\langle i|$ and *E* is a diagonal unitary matrix. The channel $\Delta$ acting on $\mathcal {A}_O \otimes \mathcal {B}_O$ is equivalent to $\Delta = \Delta _A \otimes \Delta _B$, where $\Delta _A, \Delta _B$ are completely dephasing channels acting on $\mathcal {A}_O$ and $\mathcal {B}_O$, respectively. Let us fix $\rho _*, \Omega _* \in \texttt{LOCC}$ as the optimal certification strategy achieving $\widetilde{p_{\textrm{II}}}(U, V)$. For the unitary channel certification between $\Psi _{{(UE)}^{\dag }}$ and $\Psi _{{(VF)}^{\dag }}$, where *E*, *F* are diagonal, unitary matrices, we have

20 $$\begin{aligned} p_{\textrm{II}}(\Omega _* (\Delta _A \otimes \Delta _B \otimes \mathrm{1\hspace{-0.9mm}l}_{\mathcal {A}_E \otimes \mathcal {B}_E}), \rho _*) = \widetilde{p_{\textrm{II}}}(P_U, P_V). \end{aligned}$$

Eventually, we get $p_{\textrm{II}}(UE, VF) \le \widetilde{p_{\textrm{II}}}(P_U, P_V)$, which ends the proof. $\square$

## Summary

In this study, we explored a scenario where two parties, having access to a shared quantum unitary channel, engage in its certification. Each party conducts individual measurements on their respective systems following channel utilization. We demonstrated in Theorem 1 that the certification challenge can be effectively transformed into an optimization problem involving the product numerical range. Following the proof of Theorem 1, we concluded that the optimal local strategy does not need usage of auxiliary systems and parties involved need to utilize one-way classical communication channel. We provided the original considered scheme in Fig. 1 and the optimal and resource efficient scheme in Fig. 2.

In Section 3.3 we compared local certification strategies with global ones. We observed that in the extremal case a global (single party) strategy can make no type II errors, while for the best local strategy the probability of making type II error approaches $1 - \delta$, where $\delta$ is the significance level. However, in Theorem 2 we proved that typically, for high-dimensional unitary channels, local certification strategies are optimal and what is more, they make no type II errors. Assuming Haar distribution of unitary channels of size *d*, the probability of perfect local certification is no smaller than $1 - \exp (- \frac{\log 2}{2} d)$, which is approaching exponentially fast to 1 as $d \rightarrow \infty$.

Regarding von Neumann measurements, our findings in Proposition 3 provide insights into the lower bound of the type II error, thus contributing partial but significant knowledge to the field of quantum measurement certification.

Our work opens new paths for future research. They could include more advanced comparison of *v*(*U*) and $z_{d_1:d_2}(U)$ as well as finding effective ways of computing $z_{d_1:d_2}(U)$ for arbitrary *U*. From the operational point of view, the scenario where many players are involved in the local certification process seems to be interesting to explore. As the results of our work are based strongly on LOCC measurements’ construction provided in^22^ which is also valid in many players scenario, quick analysis suggests that the Eq. (8) will be valid in that case, but with replaced

21 $$\begin{aligned} z \leftarrow \min \{|x|: x\ \in W_{d_1:\ldots :d_N}^\otimes (V^\dagger U)\}, \end{aligned}$$

where $W_{d_1:\ldots :d_N}^\otimes$ is the product numerical range defined for *N* parties^13^.

## Supplementary Information

Supplementary Information.

## Data Availability

The authors declare that the data supporting the findings of this study are available within the paper and its supplementary information files. The code used to create the plots in the Supplementary Material is available at https://github.com/iitis/NumericalShadow.jl
