# Supplementary material for: Local certification of unitary operations
Source: Sci Rep. 2024 Nov 4;14:26588. doi: 10.1038/s41598-024-75148-z (PMC11535301; doi:10.1038/s41598-024-75148-z)
Supplement: Supplementary file 1 — Supplementary Information. [file 41598_2024_75148_MOESM1_ESM.pdf]

Supplementary material: Local certification of unitary operations

Ryszard Kukulski
Faculty of Physics, Astronomy and Applied Computer Science, ul. Łojasiewicza 11, Jagiellonian University, 30-348 Kraków, Poland

Mateusz Stępniak and Kamil Hendzel
Quantumz.io Sp. z o.o., Puławska 12/3, 02-566 Warsaw, Poland

Łukasz Pawela,* Bartłomiej Gardas, and Zbigniew Puchała
Institute of Theoretical and Applied Informatics, Polish Academy of Sciences, Bałtycka 5, 44-100 Gliwice, Poland

(Dated: August 29, 2024)

I. EXAMPLES

A. Product unitary matrices

When the unitary matrix is a product $U = U_1 \otimes U_2$ (U_1 is of size d_1 and U_2 is of size d_2) we can express the minimized distance $z_{d_1:d_2}(U)$ as

$$\begin{aligned} z_{d_1:d_2}(U_1 \otimes U_2) &= \min\{|x| : x \in W^{\otimes}_{d_1:d_2}(U_1 \otimes U_2)\} \\ &= \min\{|xy| : x \in W(U_1), y \in W(U_2)\} \\ &= v(U_1)v(U_2). \end{aligned} \tag{1}$$

If $0 \in W(U_1)$ (or $0 \in W(U_2)$), then the best local and global strategies make no type II errors. Assume now, that $0 \notin W(U_1)$ and $0 \notin W(U_2)$. Let $1, \ldots, \alpha$ be the eigenvalues of U_1 ordered counterclockwise by their phases and let $1, \ldots, \beta$ be the eigenvalues of U_2 ordered counterclockwise by their phases. As zero is not in the numerical ranges of both matrices, we may write $z_{d_1:d_2}(U_1 \otimes U_2) = |1+\alpha||1+\beta|/4$. To achieve the greatest difference between local and global strategies we take $\alpha = \beta = i$. Then, 1 and $i \times i = -1$ are the eigenvalues of $U_1 \otimes U_2$, so $v(U_1 \otimes U_2) = 0$, but $z_{d_1:d_2}(U_1 \otimes U_2) = |1+i|^2/4 = 1/2$.

On the other, if $U_1 = \mathbb{1}$, then from the property of the numerical range $v(\mathbb{1} \otimes U_2) = v(U_2) = z_{d_1:d_2}(\mathbb{1} \otimes U_2)$. The value $v(U_2)$ can vary arbitrarily in the range $[0, 1]$.

B. Diagonal unitary matrices

For a diagonal unitary matrix U let us indicate its diagonal elements as $D_{i,j}$ for $i = 1, \ldots, d_1$ and $j = 1, \ldots, d_2$. We write $|\langle \psi_1, \psi_2| U |\psi_1, \psi_2\rangle| = \left|\sum_{i,j} p_i q_j D_{i,j}\right|$, where $(p_i)_i$ and $(q_j)_j$ are probability vectors that arise from diagonals of $|\psi_1\rangle\langle\psi_1|$ and $|\psi_2\rangle\langle\psi_2|$, respectively. If all eigenvalues of U are contained in an arch of length π, then one can show that to compute $z_{d_1:d_2}(U)$ it is sufficient to consider quadruples of the eigenvalues of the form $D_{i,j}$, where $i = i_1, i_2$ and $j = j_1, j_2$. In that case we optimize $|pqD_{i_1,j_1} + (1-p)qD_{i_2,j_1} + p(1-q)D_{i_1,j_2} + (1-p)(1-q)D_{i_2,j_2}|$ over $p, q \in [0, 1]$. To achieve the greatest difference between $z_{d_1:d_2}(U)$ and $v(U)$ we put $D_{1,1} = 1, D_{1,2} = D_{2,1} = i, D_{2,2} = -1$. This configuration is equivalent with product case $\{xy : x, y \in \{1, i\}\}$, which we have already solved, that is $v(U) = 0$ and $z_{d_1:d_2}(U) = 1/2$.

C. Upper-bound on z

Following the property [? , Property 9], the barycenter of the spectrum of U always belongs to the product numerical range, $\mathrm{tr}(U)/(d_1 d_2) \in W^{\otimes}_{d_1:d_2}(U)$. Hence, for any U we have

$$z_{d_1:d_2}(U) \leq \frac{|\,\mathrm{tr}(U)|}{d_1 d_2}. \tag{2}$$

D. Numerical example

Consider the family of matrices:

$$T_\alpha = (U \otimes V)^{(1-\alpha)} (\mathbb{1}_d - 2\,|\psi\rangle\langle\psi|)^\alpha, \tag{3}$$

where $|\psi\rangle = |\mathbb{1}\rangle/\sqrt{d}$ is the maximally entangled state of dimension d. and U, V are some unitary matrices. The family T_α allows us to smoothly transition from a product unitary matrix to an arbitrary one. A Monte-Carlo example of the product numerical for some values of parameter α is show in Fig. 1. Aside from showing the general shape of the numerical range the plots also show the density of points for each case.

* lpawela@iitis.pl

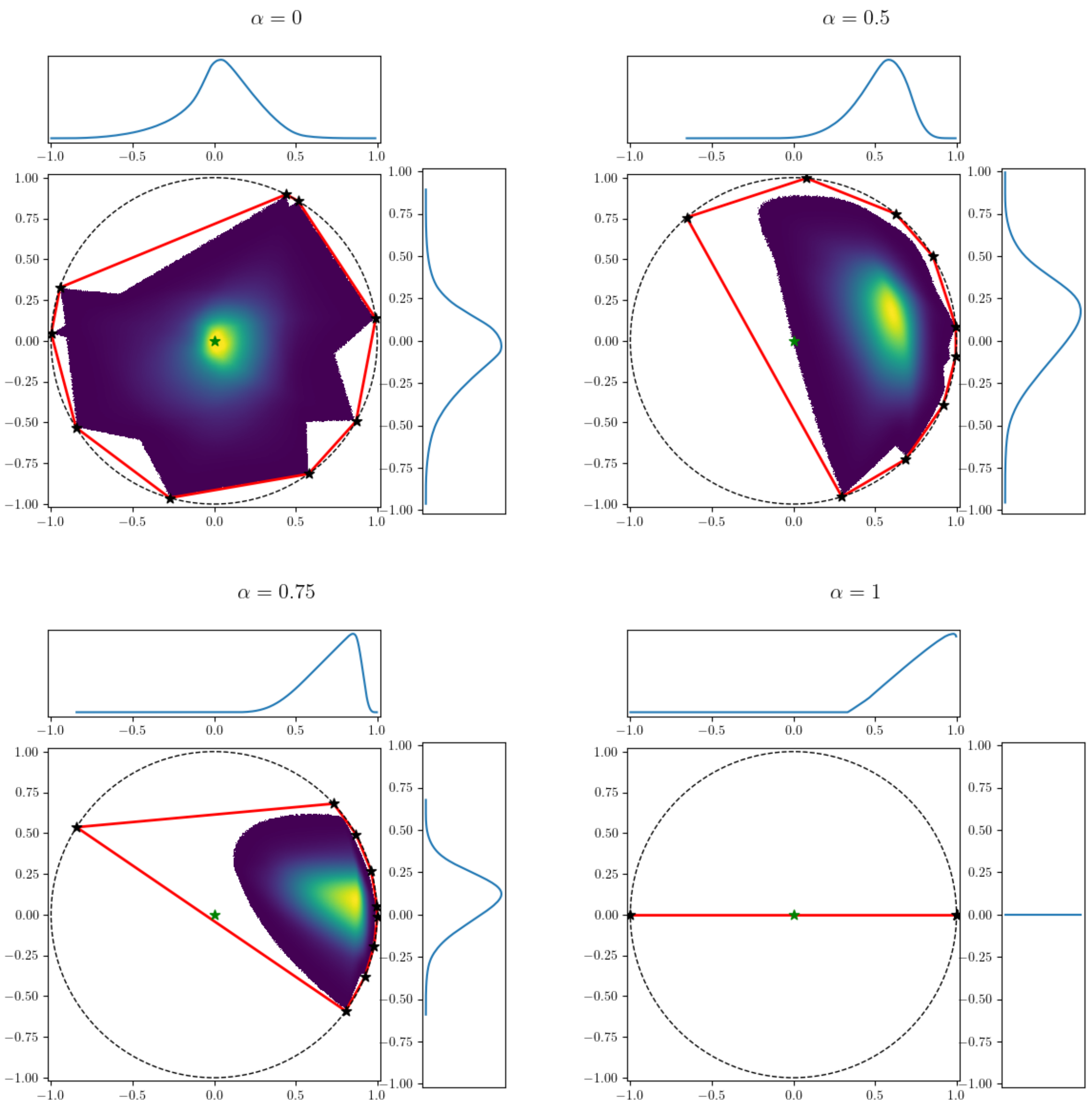

FIG. 1: Product numerical ranges of matrices from Eq. (3) for $\alpha = 0, 0.5, 0.75, 1$. The red polygon is the numerical range of T_α, the black stars are eigenvalues of T_α. The product numerical range is shown as an empirical histogram resulting from Monte Carlo simulation. The side plots are marginal distributions.
